# Sentinel lymph node biopsy in porocarcinoma: A case reports

**DOI:** 10.1016/j.ijscr.2018.10.047

**Published:** 2018-10-31

**Authors:** Simona Reina, Denise Palombo, Alexandru Boscaneanu, Nicola Solari, Sergio Bertoglio, Luca Valle, Ferdinando Cafiero

**Affiliations:** a Department of Surgery, Chirurgia I- Ospedale Policlinico San Martino Genoa, Italy; b Department of Surgical Sciences and Integrated Diagnostics (DISC) Genoa University, Italy; c Department of Anatomic Pathology, Ospedale Policlinico San Martino Genoa, Genoa, Italy

**Keywords:** Porocarcinoma, Malignant skin lesions, Sentinel node biopsy, Eccrine carcinomas

## Abstract

**Introduction:**

Eccrine porocarcinoma (EPC) is a slow-growing carcinoma arising from the eccrine sweat glands. Based on its clinical presentation it can be confused with malignant and benign skin lesions, both. Histological examination is essential to formulate a correct diagnosis. Surgical excision with clear margins is the standard therapeutic approach while the role of sentinel lymph node biopsy (SNLB) remains controversial.

**Case presentation:**

The Authors report two cases of EPC of the lower limbs occurred in two women. Patients were treated by wide surgical excision of the lesion and SNLB. 6 months follow-up was disease free for both patients.

**Conclusion:**

Although a rare cutaneous tumor, EPC has to be taken into account in the differential diagnosis of malignant skin lesions because of its possible loco-regional aggressiveness and related morbidity. Among the available treatment options, surgical excision is considered the standard approach whereas the role of SNLB is controversial although the Authors discuss a possible usefulness for staging and diagnosis.

## Highlights

• Epidemiology of rare cutaneous tumor: EPC.
• Role of Surgery excision.
• Role of SNLB for staging and diagnosis in EPC.

## 1 Introduction

Eccrine porocarcinoma (EPC), first described by Pinkus and Mehregan in 1963 [1], is a rare form of skin cancer. Its presentations very often mimics a cutaneous lesion similar to other forms of benign and malignant cutaneous neoplasms [1,2]. Accurate diagnosis, optimal treatment and prognosis of EPC are still challenging due to scant literature reports. Eccrine carcinomas may have an elevate presence of regional lymph node metastasis, thus some authors have advocated SLNB for all or some patients, but its utility for staging purposes remains unknown.

We report two cases of EPC in which the sentinel lymph node biopsy (SLNB) was performed [17].

## 2 Case presentation

### 2.1 Case 1

**Fig. 1:**
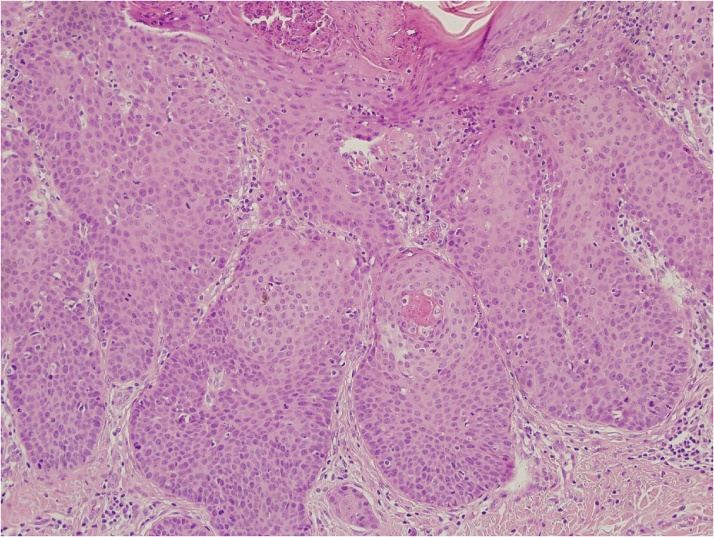
Nests of monomorphic cuboidal poroid cells with prominent nucleoli (hematoxylin & eosin stain).

During August 2017, a 64 years woman was seen at our department after a previous cutaneous lesion excision with an histological diagnosis of porocarcinoma of the left thigh. The histological examination revealed a poroid neoplasm extending into the deep dermis to the level of the dermal-subcutaneous junction with a thickness of 5.4 mm, 10–12 mitoses per 10 high-power field, absence of lymphovascular invasion and free margins with a clearing distance of 1.5 mm. Hematoxilyn-eosin staining (Fig. 1) and Immunohistochemical (IHC) analysis showed positive staining for carcinoembryonic antigen (CEA), cytokeratin (CK) 5,7 and epithelial membrane antigen (EMA).

She had a past medical history of appendicitis in childhood, anxious-depressive syndrome, osteoporosis, hiatal hernia, obesity and smoked about 20 cigarettes a day. New York Heart Association (NYHA) score was 1 and American Society of Anesthesiologists (ASA) score was 1. The patient had no anorexia and weight loss and the examination did not reveal any inguinal lymphadenopathy. Laboratory tests, including blood count, biochemical investigations and serological viral markers were normal. The electrocardiogram showed sinus rhythm and the chest radiograph showed no signs of pleural or parenchymal lesions. After multidisciplinary discussion and based on the sub-optimal clearing margin we performed a re-excision of the previous wound to ensure wider safety margins of at least 20 mm similarly to surgical strategy for other skin tumors and in particular melanoma. At that time it was also decided to perform a SLNB; preoperative lymph-node scintigraphy showed the presence of two sentinel lymph nodes in the left groin that were excised during SLNB. Recovery from surgery was uneventful and the patient was discharged on the first post-operative day. Histopathological examination found no signs of residual or satellite neoplasia in the surgical sample and the two retrieved sentinel lymph nodes were negative for metastatic disease.

Patient is disease free 7 months after the operation and continues follow- up.

### 2.2 Case 2

**Fig. 2:**
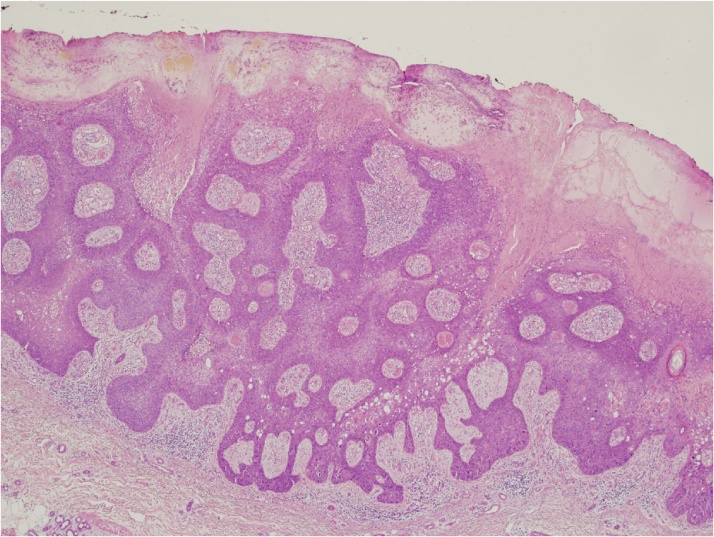
Eccrine porocarcinoma composed of basaloid cells with focal infiltration into the dermis (hematoxylin & eosin stain).

During August 2017, a 65year-old female was admitted to our department with histological finding of EPC of the right leg. One month before, she underwent surgical excision of a cutaneous lesion of the right leg. This lesion appeared brownish, exophytic, with ulcerated surface, more suggestive for a squamous cell carcinoma than an ulcerated nodular basal cell carcinoma. The histological examination revealed a poroid neoplasm extending into the reticular dermis with a thickness of 5 mm, 10 mitoses per 10 high-power field, absence of lymphovascular invasion and free margins with a clearing distance of 2 mm. Fig. 2 shows the hematoxylin-eosin stain picture of the lesion.

She had a past medical history of hysterectomy and bilateral salpingo-oophorectomy for uterine fibromatosis, kidney transplantation for severe chronic renal failure, high blood pressure, aneurysmal dilatation of the right common carotid artery, hypercholesterolemia, hyperparathyroidism and previous inferior myocardial infarction. Laboratory tests, including blood count, biochemical investigations and serum viral markers were normal. After multidisciplinary discussion and based on the sub-optimal clearing margin we performed a re-excision of the previous wound to ensure wider safety margins of at least 20 mm. It was also decided to perform a SLNB; the pre-operative lymph node scintigraphy showed the presence of two sentinel lymph nodes in the right inguinal site. The patient underwent enlargement of the surgical excision until 20 mm of free margin from the previous excision and SLNB of the two lymph nodes identified preoperatively. Recovery from surgery was uneventful and the patient was discharged from hospital on the first post-operative day. Histopathological examination found no signs of residual or satellite neoplasia in the surgical sample and the two retrieved sentinel lymph nodes were negative for metastatic disease. Patient is disease free 7 months after the operation and continues follow- up.

## 3 Discussion

EPC is a rare neoplasm arising from the intra-epidermal ductal portion of the eccrine sweat gland and represents approximately 0.005% of all cases of malignant epithelial neoplasms [[6], [7], [8],^12^].

Elderly patients are the most affected, with a peak incidence between the 6^th^ and7th decade of life. Although it does not seem to have a predilection for sex or race, some studies indicate a slight prevalence in women [7,9]. The exact etiology of EPC is unclear. Some authors suggested a possible association with radiation exposure and immunosuppression although an excessive sun exposure does not seem to be a significant risk factor [4]. EPC may arise de novo or can develop from a pre-existing benign lesion; some clinical signs, such as spontaneous bleeding, sudden growth and ulceration in a longstanding stable lesion must lead to the suspicion of malignant degeneration [10]. Clinically EPC can be presented as an erythematous or violaceous nodule, papule or plaque with an infiltrative or erosive pattern. EPC usually arises on the lower extremities (44%), followed by the trunk (24%), head & neck (23%), upper extremities (11%), and rarely involves other areas [3,5,11]. Microscopically, EPC is characterized by a cluster of anaplastic cells with nuclear hyperchromasia and important mitotic activity, extending from the epidermis to the dermis, surrounded by ductal lumen. Robinson et al. [3] reported specific histopathologic features of EPC which may be predictive of a less favorable outcome. Thickness is the main prognostic factors for EPC. Tumors greater than 7 mm in thickness, an infiltrating front of tumor cells, the presence of lymphovascular invasion, and greater than 14 mitoses per high‐power field were noted to be associated with a poorer prognosis [3].

The differential diagnosis includes basal and squamous cell carcinoma, adenocarcinoma, amelanotic melanoma, Bowen's disease, Paget's disease and also benign lesions like fibroma and pyogenic granuloma. Some immunohistochemical markers as carcinoembryonic antigen (CEA), EMA, and p53 protein may play a role in the diagnosis of EPC [13].

Therapeutic options for the treatment of EPC include electrofulguration, electrocautery, surgical excision, radiation and amputation. Surgical excision with histologically clear margins is generally considered the treatment of choice with cure rates as high as 70–80 %, although a recurrence rate of up to 20% has been reported [10]. This elevate incidence of local recurrence may be due to a not optimal free margin at surgical excision. Lymph node metastases are present at diagnosis in 20% of cases and the incidence of visceral metastases is reported to be 10% [8,9]. The tumor tends to spread tangentially in the lower third of the epidermis, then after infiltrates the dermis, subcuticular fat and lymphatic system. The role of sentinel lymph node biopsy remains controversial. Nouri et al reported a series of six patients underwent to SNLB which were negative [14]. Sahn and Lang investigated the role of SNLB in high risk EPC, they reported no positive SLNB identified among six patients but one local and one distant recurrence on follow-up [15].

Based on the observation that EPC is tumor with an important lymph node tropism some authors have suggested the possible role of SLNB for all or some EPC patients, but more studies are needed [16]. We explored SLNB approach in the two observed EPC patients with non conclusive results because in both of them retrieved sentinel nodes where shown to be free from metastasis.

## 4 Conclusions

EPC is a rare malignancy tumor; even if it can arise de novo, it often originates from the transformation of a poroma. The diagnosis of EPC should be considered in the differential diagnosis of skin lesions; early diagnosis is important because interference at an early stage could prevent the high rates of local recurrence and metastasis. Based on its low incidence rates scant protocols or guidelines are available for its diagnosis and management. In the current two case reports, we adopted SLNB as a staging and diagnostic tool similarly to other malignant neoplasms of the skin with a convergence between the non-pathological finding of the lymph nodes and the absence of disease even with a limited follow-up. Currently, the status of the SLNB affects the clinical stage of patients with EPC but its prognostic or potential therapeutic role remain to be determined with Larger studies and extended follow-up.

## Conflict of interest

None.

## Sources of funding

None.

## Ethical approval

The paper is not a research study and is not require a ethical approval in our institute.

## Consent

Written informed consent was obtained from the patient for publication of this case report.

## Author contribution

**Simona Reina**, ideated the study and drafted the article.

**Denise Palombo**, substantial contributions to conception and design.

**Alexandru Boscaneanu**, acquisition of data.

**Nicola Solari**, revising it critically for important intellectual content.

**Sergio Bertoglio**, revising it critically for important intellectual content.

**Luca Valle**, acquisition of data.

**Ferdinando Cafiero**, final approval of the version to be submitted.

All authors approved the final draft.

## Registration of research studies

Our research was recorded on Clinica trial.gov.

The clinical trial identifier number is NCT03647631.

Other study ID number: Chirurgia1.

## Guarantor

Dr.ssa Simona Reina. Department of Surgery, Chirurgia 1 Ospedale Policlinico San Martino, Padiglione 15, III piano ponente - Largo Rosanna Benzi, 10 16132 – Genova, Italia. Tel. 3492634555.

E-mail: simona.reina10@gmail.com.

## Provenance and peer review

Not commissioned, externally peer reviewed.
